# Elucidation of Functional Roles of Sialic Acids in Cancer Migration

**DOI:** 10.3389/fonc.2020.00401

**Published:** 2020-03-31

**Authors:** Huiyan Sun, Yi Zhou, Hongyang Jiang, Ying Xu

**Affiliations:** ^1^Cancer Systems Biology Center, The China-Japan Union Hospital, Jilin University, Changchun, China; ^2^School of Artificial Intelligence, Jilin University, Changchun, China; ^3^Computational Systems Biology Lab, Department of Biochemistry and Molecular Biology and Institute of Bioinformatics, the University of Georgia, Athens, GA, United States

**Keywords:** sialic acids, cancer migration, transcriptomic data, electrostatic repulsion, metastasis

## Abstract

Sialic acids (SA), negatively charged nine-carbon sugars, have long been implicated in cancer metastasis since 1960's but its detailed functional roles remain elusive. We present a computational analysis of transcriptomic data of cancer vs. control tissues of eight types in TCGA, aiming to elucidate the possible reason for the increased production and utilization of SAs in cancer and their possible driving roles in cancer migration. Our analyses have revealed for all cancer types: (1) the synthesis and deployment enzymes of SAs are persistently up-regulated throughout the progression for all but one cancer type; and (2) gangliosides, of which SAs are part, tend to converge to specific types that allow SAs to pack at high densities on cancer cell surface as a cancer advances. Statistical and modeling analyses suggest that (i) a highly plausible reason for the increased syntheses of SAs is to produce net protons, used for neutralizing the OH^−^ persistently generated by elevated intracellular iron metabolism coupled with chronic inflammation in cancer tissues; (ii) the level of SA accumulation on cancer cell surface strongly correlates with the stage of cancer migration, as well as multiple migration-related characteristics such as altered cell-cell adhesion, mechanical stress, cell protrusion, and contraction; and (iii) the pattern of SA deployment correlates with the 5-year survival rate of a cancer type. Overall, our study provides strong evidence for that the continuous accumulation of SAs on cancer cell surface gives rise to increasingly stronger cell-cell repulsion due to their negative charges, leading to cell deformation by electrostatic force-induced mechanical compression, which is known to be able to drive cancer cell migration established by recent studies.

## Introduction

It has been observed that increased syntheses of sialic acids (SAs) are associated with cancer development and metastasis since 1960's (1, 2), where SAs are negatively charged nine-carbon sugars and generally serve as the capping molecules of cell-surface glycan, as part of plasma membrane-embedded gangliosides (3). Under physiological conditions, brain tissues have the highest concentration of SAs, used for synaptogenesis. Outside of brain, red blood cells have the highest cell-surface concentration of SAs. As of now, it remains largely unknown of why cancer cells produce unusually large numbers of SAs and accumulate them on cell surface (4). Published studies have been mostly focused on their signaling roles with other cell types such as immune and endothelial cells, via binding to siglecs and selectins, to facilitate interactions between cancer and immune cells (5) and to enable cancer cells interactions with and penetration into blood vessels (1), respectively. Very little has been established regarding if they may play roles in creating mechanical compression within cancer tissues, knowing their negative charges and being increasingly placed on cell surface, hence possibly resulting in increasingly stronger cell-cell repulsion, while mechanical pressure has been widely observed in cancer tissues but largely attributed to the confined space for growing tumors (6, 7).

We have recently studied 44 reprogrammed metabolisms widely observed in cancer, including persistent SA synthesis, and discovered that each of them produces more protons than its original metabolism (8). In addition, we have also discovered that all cancer tissue cells harbor Fenton reactions: Fe^2+^ + H_2_O_2_ -> Fe^3+^ + ·OH + OH^−^ in their cytosol; and the rates of OH^−^ production can saturate the intracellular pH buffer within days, hence increasing the intracellular pH if not neutralized (9), which posts a major stress to the host cells. A linear regression analysis was conducted of the predicted level of Fenton reactions against the predicted levels of all ~50 reprogrammed metabolisms (Figure S1), which achieves high *R*^2^ values with statistically significant *p*-values for each cancer type (8). This strongly suggests that these reprogrammed metabolisms are induced to respond to cytosolic Fenton reactions, serving as neutralizers for the OH^−^ persistently produced by Fenton reactions.

In this context, we present a computational study of transcriptomic data of SA related vs. migration related genes in cancer tissues of eight cancer types from the TCGA database (10). Our analyses have revealed: (1) majority of the cancer types has increased production and deployment of SAs on cell surface, where the synthesis of a SA generates two net protons; (2) the level of SA synthesis correlates with the level of cytosolic Fenton reaction for all cancer types studied; and (3) as a cancer progresses, it tends to converge to use of specific types of gangliosides, facilitating SA packing at high densities. Further analyses lead to the following discoveries: (i) a simple model for predicting the level of SA accumulation on cell surface can statistically well explain cancer progression from stage N0 through stage N3 and then stage M (using the TNM stage notation), where Ni represents cancer tissues that have metastasized to i lymph nodes and M for distant metastasis; (ii) strong correlations are observed between a range of cell migration-associated characteristics, such as increased mechanical stress, cell contraction and protrusion, and SA production and/or deployment; and (iii) the detailed expression patterns of SA synthesis and degradation genes can statistically well explain the average 5-year survival rates of each cancer type, hence the level of metastasis since the survival rate strongly correlates with the level of metastasis across all eight types. Overall, our analyses provide strong evidence for that the SA accumulation on cancer cell surface plays key roles in mechanical compression and cell deformation in cancer tissues.

It has been observed that cancer tissues have strong mechanical pressure within (6, 7). It was suggested that this is due to the confined space for the growing tumors. However, the “confined space” argument may not be supported by experimental data. For example, skin melanoma starts to metastasize as soon as the cancer starts to grow vertically, which is clearly not confined by space. Our analyses suggest: mechanical pressure strongly correlates with the cell-surface accumulation level of SAs. A previous study has convincingly demonstrated that mechanical compression can lead to cell deformation, which can drive the activation of actomyosin filaments and associated contractile motion, ultimately driving collective migration by cell clusters with enhanced cell-cell adhesion (11).

By integrating all these together, we have developed a model for how SA synthesis and deployment, responding to cytosolic Fenton reactions, can give rise to increasingly stronger cell-cell repulsion, further leading to mechanical compression and cell deformation, which can drive cancer cell migration.

## Results

### Elevated Synthesis of Sialic Acids in Cancer

**Figure 1:**
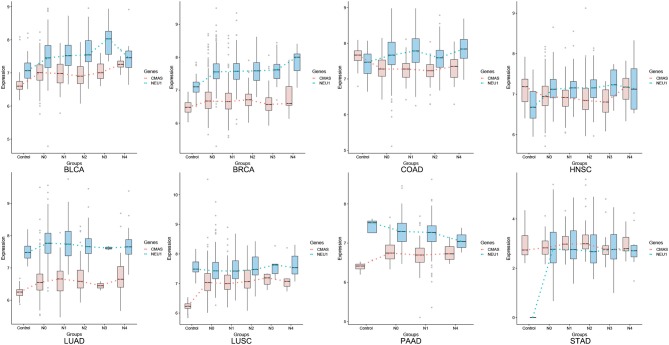
Average expression levels (y-axis) of CMAS (SA synthesis, blue) and NEU1 (SA degradation, orange) in tissues of cancer adjacent control, stage N0, N1, N2, N3, and M (x-axis), respectively.

We have examined the key genes involved in SA synthesis (CMAS) and degradation (NEU1). CMAS is up-regulated in seven of the eight cancer types (except for COAD); and NEU1 tends to correlate with CMAS throughout the major portion of a cancer progression for all cancer types, except for the last stage(s), where the two curves may diverge or converge for some cancer types, as detailed in Figure 1.

Previous studies generally attribute the increased SA production to their signaling roles in cancer (12). However, this is not supported by the transcriptomic data analyzed here. It is known that SAs conduct their functions through binding with the siglec and/or selectin molecules. Our analyses of the gene expression data in (bulk) cancer tissues show that there is no or little correlation between CMAS and any siglec gene (SIGLEC1-16), selectin gene (SELE, SELL, and SELP) or their total expressions (data not shown).

We have previously predicted that 44 known reprogrammed metabolisms (8), including persistent production of SA, in cancer are induced to generate protons for neutralizing OH^−^ persistently generated by cytosolic Fenton reactions.

**Figure 2:**
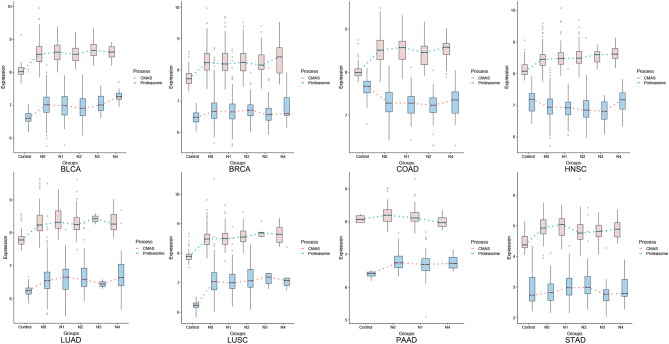
Predicted levels of cytosolic Fenton reactions (orange) vs. expression levels of CMAS gene (blue), where the level of Fenton reaction is estimated based on the expressions of genes related to macromolecular damages such as proteasome genes, as given in Sun et al. (9).

Figure 2 shows the predicted levels of cytosolic Fenton reactions (9) vs. the expression level of CMAS across different stages of all cancer types under study. The majority of the cancer types show statistically significant positive correlations like BRCA (cor = 0.352, *p*-value = 3.05E-33), COAD (cor = 0.216, *p*-value = 2.32E-04), LUAD (cor = 0.343, *p*-value = 1.04E-15), LUSC (cor= 0.206, *p*-value = 3.45E-06), STAD (cor = 0.212, *p*-value = 9.96E-04). While the detailed correlation between the two curves may not always be high due to contributions by other reprogrammed metabolisms to neutralize OH^−^ by Fenton reactions, their overall trends are generally the same. Hence, we predict: the SA synthesis is induced to respond to cytosolic Fenton reactions.

### Accumulation of Sialic Acids on Cancer Cell Surface and 5-Year Survival

It has been established that cancer cells accumulate SAs on cell surface, and the level of such accumulation could be considerably higher than that of red blood cells (13), which are known to have high-levels of cell-surface SAs that prevent red blood cells from adhering to each other due to their negative charges (14). If our immune system detects adhered red blood cells, it will destroy them as their adhesion indicates that such cells have reached the end of their working lives.

Knowing that the accumulation rate of SAs depends on the rates of SA synthesis, degradation, and transfer to cell-surface glycan, we have used the expressions of the following genes to estimate the rate of such accumulation. CMAS is used for SA synthesis, NEU1 for its degradation, and sialyltransferase genes ST3GAL1, 2, 5, ST6GALNAC4, 5, and ST8SIA1, 5 for SA transfer to glycan. The following is used to estimate the rate of the SA placement onto glycan:

$$\begin{array}{l} \sqrt{E\left(CMAS\right)*\sum_{Y}E\left(Y\right)} \end{array}$$

where *E(X)* is for the expression of gene X, and Y represents the seven SA transferase genes. Similarly, the following is used to estimate the rate of SA degradation, where CTSA (Cathepsin A) is needed to form a complex with NEU1 to conduct the degradation function.

$$\begin{array}{l} \sqrt{E\left(NEU1\right)\;*\;E\left(CTSA\right)} \end{array}$$

**Figure 3:**
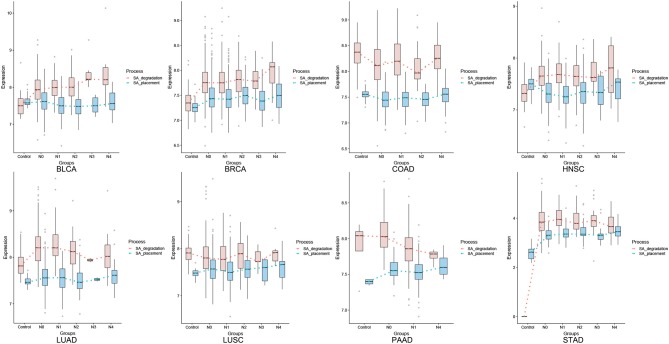
Expression levels for SA placement on cell surface (blue) and degradation (orange).

Figure 3 shows the comparative levels of these two quantities across different stages of all eight cancer types. An assumption used here is: for a gene X with expression level *E*(X), the maximum reaction rate of the enzyme encoded by X is proportional to K_cat_ * *E*(X), with K_cat_ being the reaction rate constant catalyzed by enzyme X in the Michaelis-Menten formulation [NOTE: this is essentially equivalent to the assumption that (i) the expression level of a gene is linearly proportional to its protein concentration; and (ii) the reactant concentration is higher than the reaction constant K_M_, a common assumption used when modeling human metabolisms based on Michaelis-Menten formulation]. Hence the reaction rates of the SA placement and degradation should be linearly proportional to the two curves for each cancer in Figure 3. Knowing that CMAS and NEU1 have comparable K_cat_ values (15, 16), we predict that the two curves reflect the relative reaction rates of the two enzymes.

**Figure 4:**
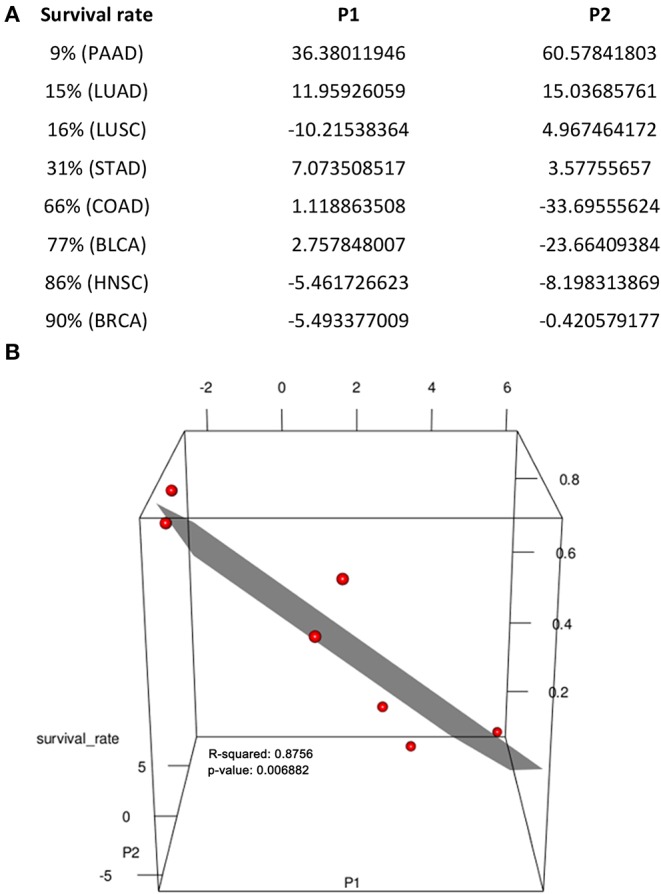
Regression of 5-year survival rate against sialic-acid related gene expression data. **(A)** Parameters used. **(B)** Visualization of data in **(A)** in 3D space, where each red dot represents one line in **(A)**, achieving *R*^2^ = 0.876, *p*-value = 6.882E-03 when excluding LUSC, which does not fit our model.

From Figure 3, we conclude: (1) pancreatic cancer (PAAD) has by far the largest (positive) difference between the SA placement and degradation, measured using the total area between the curves of SA placement and SA degradation (the area is negative if the degradation curve is above the placement curve), hence giving rise to highest level of the SA accumulation and the strongest cell-cell repulsion, which is consistent with the known fact that the cancer has the highest death rate, among all cancer types; and (2) more generally, cancer types with higher 5-year survival rates tend to have higher SA degradation rates than their placement rates, especially toward the last stage of a cancer. Figure 4 summarizes the average 5-year survival rates of the eight cancers under study.

To further demonstrate that the rates of the SA placement and degradation may have implications to a cancer's 5-year survival rate, we have conducted a linear regression analysis of the survival rate against changes in the rate of the SA placement and the rate of its degradation in the last two stages for each caner type. Specifically, let P1 be the difference between the slopes of the SA placement curve and the slopes of SA degradation in the last two stages and P2 be the difference between the values of SA placement curve and degradation in the last stage of each cancer (Figure 3). Figure 4 shows a visualization of the values of these two parameters along with the corresponding survival rate for each cancer type. We can see that the survival rate can be well explained by these two parameters achieving *R*^2^ = 0.876, *p*-value = 6.882E-03, when not considering LUSC, which does not fit our model. Hence, the regression analysis is conducted only on the other seven cancer types, which gives the following function and achieves a high fitness level *R*^2^ = 0.876 and *p*-value is 6.882E-03.

$$\begin{array}{l} S=0.63-0.0725\;\times \;\mathrm{sign}\left(P1\right)\sqrt{\left|P1\right|}-0.2279\;\times \;\mathrm{sign}\left(P2\right)\sqrt{\left|P2\right|}\;. \end{array}$$

### Ganglioside Types as a Cancer Advances

Under physiological conditions, gangliosides, as the hosts of SAs, are predominantly used in brains and red blood cells. In the embryonic stage, brain cells tend to use simple gangliosides, i.e., those with simple glycan structures and gradually switch to more complex structures, such as GM1, GD1a, GD1b, and GT1b (17), where GM is for monosialoganglioside, GD for disialoganglioside and so on.

It has been observed that advanced stage cancers tend to use specific gangliosides such as GD2 and GM2, or GD3 and GM3 to a lesser extent (18). Majority of the published studies focus on the possible signaling roles of such gangliosides like GD2 or GM2 (19, 20). Other authors have examined the issue from the perspective that different gangliosides enable different packing densities of gangliosides inside plasma membrane, and observed: generally, the simpler a glycan structure, the more gangliosides can be packed into a fixed area.

**Figure 5:**
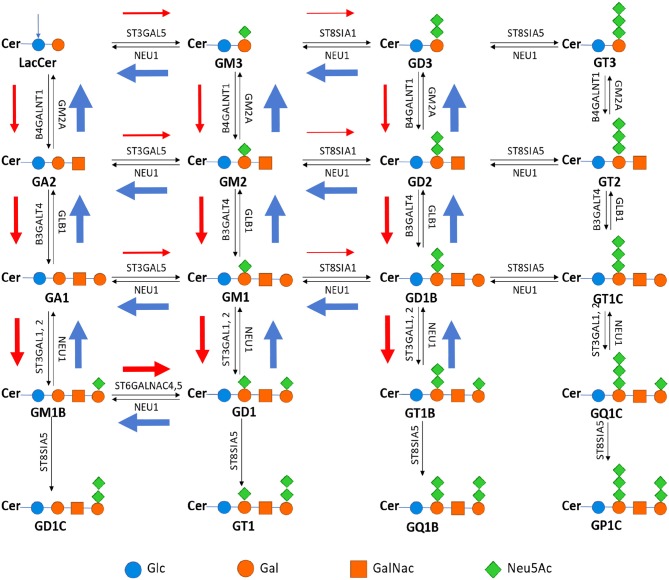
Metabolic pathway of ganglioside synthesis and metabolism, where the name in bold is the name of the ganglioside above it; and the name next each arrow represents the gene encoding the enzyme that catalyzes the reaction represented by the arrow [adapted from Yu et al. (21)]. The width of each red/blue arrow represents roughly the relative expression level of the corresponding gene, and color red is for reactions that each produce one net proton and blue for pH neutral reactions while reactions without such arrows are for those without expressions.

To understand these observations, we have examined the expression data of the enzyme genes in the synthesis network of gangliosides (Figure 5), where the “typical” relative expression levels of these genes are shown across the eight cancer types. We note: the synthesis pathways of numerous gangliosides do not quite follow the normal pathways as shown in Figure S2, instead they form distinct synthesis fluxes as shown in Figure 5, shown by the red/blue arrows with different widths. Specifically, the flux first goes downwards along the first column, and then travels to the second column via the ST6GALNAC4/5-catalyzed reactions more than the typical ST3GAL5 reactions. The flux then travels upwards along the second column; and from the second to the third column, the flux is relatively weak via the moderately expressed ST8SIA1.

We have then examined the total expression level of the influx enzymes for each ganglioside (i.e., those that produce the ganglioside) vs. that of the efflux enzymes (i.e., those that utilize the ganglioside); and predict that a ganglioside will have cellular accumulation if its influx rate is higher than its efflux rate, otherwise the ganglioside will not be accumulated. Note that such accumulation of a ganglioside should be proportional to the level of its deployment inside the plasma membrane. The reason we do this simplified qualitative network flux analyses, instead of the detailed kinetics-based Michaelis-Menten analyses is: we do not have the kinetic rate constants, K_cat_ and K_M_, for multiple enzymes under consideration.

This analysis has revealed that advanced-stage cancer tissues generally have high accumulations of GM2 and GD2, followed by GM3 and GD3 (Table 1). These results are highly consistent with the published results.

**Table 1:** Estimated accumulation level of gangliosides across different stages of cancer metastasis.

**Cancer type**	**Early stage (N0)**	**Mid stages (N1, 2,3)**	**Late stage (M)**
BLCA	GM2 (high), GM3 and GD3 (moderate)	GM2 (high), GD3 (moderate)	GM2, GD2 (high), GM3, GM1 (moderate)
BRCA	GM2 (high), GD3, GD2, GD1 (moderate)	GM2 (high), GD3, GD2, GD1 (moderate)	GM2 (high), GD3, GD2 (moderate)
COAD	GM2, GD2 (high)	GM2, GD2 (high)	GM2, GD2 (high)
HNSC	GD3 (high), GM2, GD2 (moderate)	GD3 (high), GM2, GD2 (moderate)	GD3 (high), GM2, GM1, GD2 (moderate)
LUAD	GM3, GM2, GM1 (high), GD3, GD2, GD1 (moderate)	GM3, GM2, GM1 (high), GD3, GD2, GD1 (moderate)	GM3, GM2, GD1 (high), GM1, GD3 (moderate)
LUSC	GM2 (high), GD3, GD2 (moderate)	GM2 (high), GD3, GD2 (moderate)	GM2 (high), GM1, GD3, GD2 (moderate)
STAD	GM2, GD1 (high), GD2 (moderate)	GM2, GD1 (high), GD2 (moderate)	GM2, GD1 (high), GD2 (moderate)
PAAD	GM2, GD1 (high), GM1B, GD2 (moderate)	GM2, GD1 (high), GD2 (moderate)	GM2 (high), GD2, GD1 (moderate)

A key discovery made in our previous work on cancer metabolic reprogramming is: cancer tends to produce as many protons as possible in each reprogrammed pathway; and the altered ganglioside synthesis processes is one of the reprogrammed metabolisms studied (8). Hence, we hypothesize: cancer may select specific ganglioside types that maximize the total number of protons produced per cell plasma membrane.

To examine if this is the case, we have examined the packing densities of GM2 and GM1. It has been reported that 451 GM2 molecules pack into a cluster with head (cross-section) radius 66.0 Å and 301 GM1 form a cluster with head radius 58.7 Å (22), hence the ratio between the head areas per GM1 and GM2 is 58.7^2^/301: 66^2^/451, namely 11.45: 9.66. Therefore, for a fixed area, the ratio between the numbers of GM1 and GM2 that can pack into is: 9.66: 11.45.

Note that the normal pathways for synthesizing GM1 and GM2 produce 3 and 2 net protons (the numbers next to the red arrows along the pathway), respectively, but the altered pathways each produce 4 protons. In addition, the synthesis of each ganglioside requires the synthesis of some SAs, each of which produces two net protons. Figure 5 shows the number of SAs attached to each ganglioside. Hence for the same area in plasma membrane, the ratio between the numbers of protons that the syntheses of GM1 and GM2 each produce is: 9.66 x (4 + 2): 11.45 x (4 + 2), namely 9.66: 11.45, respectively. Therefore, more protons are produced by the synthesis and utilization of GM2 than that of GM1.

Considering that no published data regarding the packing densities in the same setting for the other gangliosides are publicly available (to the best of our knowledge), we extrapolate that GM3 and GM2 have a similar relationship to that between GM2 and GM1 presented above. Hence we predict that maximizing the proton production is a key determinant in a cancer's selection of utilization frequencies of GM3, GM2, and GM1 as observed above.

### A Sialic Acid-Based Model for Cancer Metastasis

Under the TNM scheme, a cancer tissue is classified into stage N0, N1, N2, N3, or M, where Ni is for tissues with i nearby lymph node(s), 0 ≤ *i* ≤ 3, being metastasized and M is for distant metastasis. In the following, N4 represents M for the simplicity of presentation. Our goal is to demonstrate that for each cancer type, the average SA accumulation level in stage Ni, 0 ≤ *i* ≤ 4, can be calculated as ℂ_*i*_+ C_i_, where ℂ_*i*_ denotes the SA level accumulated solely in stage Ni and C_i_ is a fixed positive value denoting the SA level accumulated in the previous i-1 stages for i ≥1 and C_0_ = 0. Furthermore, ℂ_*i*_ is a function of the rates of SA synthesis, degradation and transfer to cell-surface glycan, respectively, and the duration of stage Ni, which can be estimated based on the expression data of seven genes: CMAS for SA synthesis, NEU1 for its degradation, ST3GAL1, 2, 5 and B4GALNT1 for its transfer onto cell-surface glycan, and POLDIP2 (a DNA polymerase gene) for the rate of cell cycle whose inverse is a proportional to the duration of one cell cycle. Mathematically, this problem can be formulated as: for each cancer type, search for an unknown function *F*():${\;\mathbb{R}}^{7}\to \left\{{\mathbb{N}}_{i}\right\}$ and an unknown fixed positive value *C*_*i*_, with ℕ_*i*_ being set to the following values: ℕ_*c*_ = 0, ℕ_0_ = 0, ℕ_1_ = 1, ℕ_[2, 3]_ = 2, ℕ_4_ = 3 so

$$\begin{array}{l} \underset{F,C_{i}}{\text{min}}\;\|{\mathbb{N}}_{i}-\;F(E(\text{CMAS}),\;E(\text{NEU1}),\;E(\text{STA3GAL1}),\\ \;E(\text{STA3GAL2}),\;E(\text{STA3GAL5}),\;E(\text{B4GALNT1}),\\ \;E(\text{POLDIP2}),\;C_{i})\|\; \end{array}$$

over all samples in stage Ni, i = C, 0, 1, [2,3], 4, where Nc is for control samples; N[2,3] is the union of samples in N2 and N3, each of which tends to be too small, hence combined; and E(X) is the expression value of gene X in a sample under consideration.

**Figure 6:**
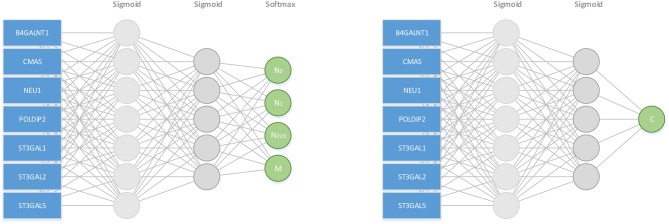
A neural net based model for predicting the stage of metastasis using SA synthesis, degradation and deployment genes and cell cycle-related gene: CMAS, NEU1, ST3GAL1, 2, 5, B4GALNT1, and POLDIP2.

This problem is solved as a multi-classification and a regression problem using two separate neural networks, each with two-hidden layers, one for evaluating the performance of multi-classification model, and on this basis, the other for solving the Ci values and finding the F() function, respectively. Figure 6 shows the two neural network architectures. In the left panel of Figure 6, for each cancer type and each stage Ni, 70% samples are randomly selected as training data and the remaining as the testing data, and this process is repeated for 10 times. Table 2A shows the prediction results for each stage Ni and each cancer type, measured using the macro F1 score, defined as:

$$\begin{array}{l} F_{1}=2\frac{MR\;\times MP}{MR+MP} \end{array}$$

where $MP=\overset{n}{\underset{i}{\sum }}\frac{TP_{i}}{TP_{i}+FP_{i}}$, $MR=\overset{n}{\underset{i}{\sum }}\frac{TP_{i}}{TP_{i}+FN_{i}}$, TP_i_, FP_i_, and FN_i_ are rates of true positive, false positive and false negative for predicting the samples of the ith stage, respectively.

**Table 2:** Prediction accuracy of metastasis stage.

**(A) Macro average F1 score of multi-classification for each cancer**.				
**Cancer type**		**Macro average F1 score**		
BLCA		0.744		
BRCA		0.555		
COAD		0.765		
HNSC		0.649		
LUAD		0.638		
LUSC		0.739		
PAAD		0.794		
STAD		0.772		
**(B) Estimation of the SA accumulation level prior to stage Ni**.				
**Cancer type**	**C**_**0**_	**C**_**1**_	**C**_**[2, 3]**_	**C**_**4**_
BLCA	16.340	18.090	26.847	58.350
BRCA	18.862	21.054	25.988	54.064
COAD	13.218	22.007	33.283	51.469
HNSC	16.701	19.715	23.493	59.880
LUAD	14.238	19.516	32.094	54.725
LUSC	14.042	19.488	26.718	59.526
PAAD	15.532	24.576	NA	60.030
STAD	11.895	17.719	30.342	59.992

In the right panel of Figure 6, the loss function of the neuron network is $\min\overset{n}{\underset{i=1}{\sum }}{\left(y-\hat{y}\right)}^{2}$, where *ŷ* is the predicted c value. When the maximum number of iterations reached 1,000,000 or the loss is less than 0.1, the training process is done. Table 2B summarizes the predicted Ci values for each cancer type, which increase monotonically over stage, hence logically meaningful.

From the table, we conclude that for each cancer stage, the seven genes can statistically well explain the metastasis stage, hence strongly suggesting that the SA accumulation level on cancer cell surface is a key factor in dictating the level of metastasis of a cancer tissue.

#### Supporting Evidence

To provide further evidence that the SA accumulation on cancer cell surface can strongly influence the level of metastasis of a cancer, we have examined the statistical relationships between the above seven genes and a range of characteristics uniquely associated with cancer migration, each of which is reflected by a set of marker genes given in Table 3. For a given set of marker genes {g_1_, …, g_k_} over *n* samples, let X = (x_1_, …, x_n_)^T^ be the solution that minimizes the following function:

$$\begin{array}{l} \left|\frac{1}{n}\times \left(\begin{array}{c} \underset{1\leq i\leq n}{\sum }E_{i}\left(g_{1}\right)\\ \ldots .\\ \underset{1\leq i\leq n}{\sum }E_{i}\left(g_{k}\right) \end{array}\right)\;-\;\left(\begin{array}{ccc} E_{1}\left(g_{1}\right) & \ldots & E_{n}\left(g_{1}\right)\\ .. & .. & ..\\ E_{1}\left(g_{k}\right) & \ldots & E_{n}\left(g_{k}\right) \end{array}\right){\left(x_{1},\;\ldots ,\;x_{n}\right)}^{T}\right|\; \end{array}$$

where *E*_*i*_(*g*_*j*_) is the expression level of gene *g*_*j*_ in sample I, and X represents the feature vector of {g_1_, …, g_k_} over the n samples, which is used to calculate the co-expression level with the SA related genes. This problem is solved as a linear regression problem.

**Figure 7:**
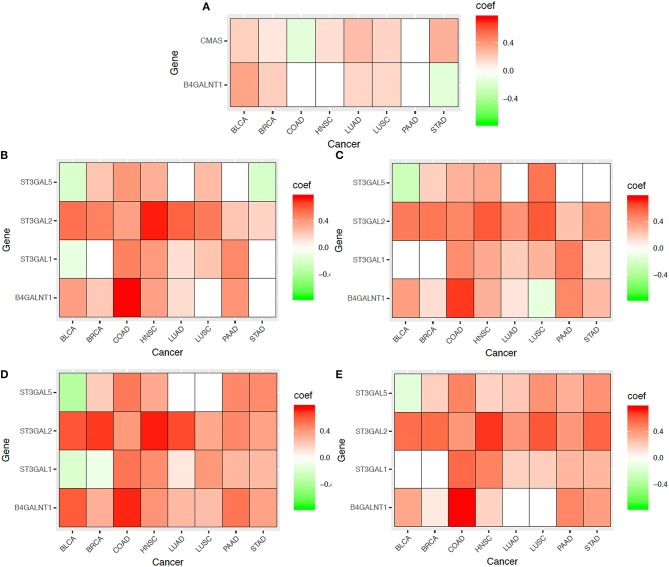
Co-expressions between SA related genes (CMAS, ST3GAL1, ST3GAL2, ST3GAL5, B4GALNT1) and marker genes (as shown in Table 3) for multiple aspects of cancer metastasis: **(A)** Mechanical compression genes, **(B)** Cell-cell adhesion genes, **(C)** Cell contraction genes, **(D)** Protrusion genes, and **(E)** Motion/migration genes across eight cancer types. In the heatmaps, the red represent positive correlation, the green represent negative correlation and the white represent insignificant correlation respectively.

- Mechanical compression marker genes and SA related genes: We have examined co-expression levels between the above SA related genes and known marker genes of compressive stress. Figure 7A shows that compressive stress marker genes strongly correlate with the SA genes. We have then conducted a regression analysis of the marker genes against the SA genes, with regression results shown in Table 4.
- Cell-cell adhesion genes and SA related genes: It has been known that cancer cells tend to alter their cell-cell adhesion genes as cancer cells start to migrate. The co-expression data and regression results are given in Figure 7B and Table 4.
- Cell contractile genes and SA related genes: The co-expression data and regression results are given in Figure 7C and Table 4.
- Protrusion genes and SA related genes: The co-expression data and regression results are given in Figure 7D and Table 4.
- Motion marker genes and SA related genes: The co-expression data and regression results are given in Figure 7E and Table 4.

From these figures and tables, we can see that each of the key migration-related characteristics can be well explained statistically by the SA genes, hence providing further evidence to that SA accumulation plays key roles in driving cancer migration.

**Table 3:** Marker genes for processes related to cancer cell migration.

**Migration related process**	**Marker genes**
Mechanical stress	CASP3, DSG1, DSG2, DSG3
Cell-cell adhesion	CDH11, CDH13, CDH1, CDH2, CDH3, CDH5, CDH24, CTNNBL1, CTNND1, CTNNB1
Cell contraction	PTK2B, PDCD10, KCTD13, ITGB1, PHACTR1, CUL3, SRC, SRF, ARRB1, RHOA, SORBS1, TNFAIP1, ZYX, ITGB5
Protrusion	CENPB, RAB13, ZEB1, ANP32B, CORO1A, PINK1, DCC, VLDLR, FSCN1, TIAM1, MAP1B, LRP8, RELN, DCX, DCLK1, GAP43, FEZ1, CXCR4, DBN1, CTTN, ARP2, ARP3, CFL1, CFL2, LIMK1, LIMK2, WASF1, WASF2
Motion and migration	AMOT, FGF2, GPLD1, GPR124, KDR, NRP1, PTK2B, SCARB1, TDGF1, VEGFA

**Table 4:** Regression results of migration-related characteristics against SA related genes.

	**BLCA**	**BRCA**	**COAD**	**HNSC**	**LUAD**	**LUSC**	**PAAD**	**STAD**
Cell-cell adhesion	0.562	0.495	0.797	0.754	0.581	0.529	0.578	0.299
Polarity	0.464	0.261	0.667	0.425	0.192	0.368	0.358	0.271
Mechanical stress	0.508	0.365	0.199	0.173	0.368	0.367	0.437	0.471
Protrusion	0.730	0.697	0.792	0.775	0.666	0.532	0.638	0.566
Cell contraction	0.555	0.513	0.720	0.631	0.444	0.705	0.623	0.412
Motion and migration	0.553	0.557	0.810	0.759	0.464	0.646	0.536	0.667
***p*****-values of the calculated** ***R***^**2**^ **values**								
Cell-cell adhesion	6.28E-33	7.62E-66	4.51E-61	1.60E-93	2.38E-45	4.96E-35	2.33E-15	3.33E-05
Polarity	4.02E-21	1.05E-16	1.50E-35	3.99E-22	1.18E-04	1.08E-15	1.39E-05	2.20E-04
Mechanical stress	5.63E-26	1.34E-33	4.12E-03	5.82E-04	3.76E-16	1.23E-15	2.96E-08	7.56E-13
Protrusion	2.72E-66	2.59E-156	1.25E-59	6.75E-102	1.68E-64	1.30E-35	1.24E-19	1.68E-19
Cell contraction	5.93E-32	1.27E-71	3.18E-44	2.92E-56	3.33E-24	1.29E-73	1.73E-18	1.15E-09
Motion and migration	9.10E-32	2.14E-87	2.21E-64	3.26E-95	1.05E-26	7.82E-58	6.81E-13	1.41E-29

### Regulation of Key Genes Leading to Cancer Migration

We have conducted an integrated analysis of genomic, epigenomic, and transcriptomic data to estimate how the key SA genes, namely CMAS, ST3GAL1, ST3GAL5, and NEU1, are regulated across different cancer types. For each gene, we assess the level of contribution to its transcription regulation by transcription factors vs. DNA methylation using a regression analysis (see section Methods).

Table 6 shows the detailed regression results for all four genes. From the table, we can see (1) more DNA methylation utilization in cancer samples than in normal samples; and (2) while the level of contribution to transcription regulation by DNA methylation varies across different genes and different cancer types, DNA methylation in general makes substantial contributions to the regulation of the key SA genes.

## Discussion

Our prediction for linking overproduction and gradual accumulation of sialic acids to cancer migration is based on identified connections, statistical or physical, among seemingly unrelated molecular species and cellular activities. It is the multiplicity of such chains of simple and subtle associations, which are otherwise do not exist, that give us the statistical confidence about our prediction. Some of the detected associations are only useful for making our overall prediction while others, such as the 7-gene signature for predicting the status of cancer metastasis, could be used independently as novel markers for cancer metastasis. In addition, our prediction confidence also comes from the previous work that causally connects mechanical forces to cancer migration.

Tse et al. (11) presented an elegant study that shows external mechanical compression on cancer cells can lead to their deformation, which can give rise to enhanced cell-cell adhesion, actomyosin contraction, filopodial protrusion, ultimately collective migration by clusters of cells. This model provides strong support to our prediction that SA accumulation on cancer cell surface will generate increasingly stronger electrostatic repulsion due to the increased densities of electric charges from SAs, leading to enhanced cell-cell adhesion, actomyosin contraction, protrusion as well as migration as established above. Compared to previous studies on SAs and cancer migration, our study provides a fundamentally novel perspective regarding the roles of SA in cancer migration, namely it is their physical property rather than the signaling functions that may play the predominant role in driving cancer metastasis.

This model can answer a few long standing open questions regarding cancer metastasis: (1) while SAs have long been known to be associated with cancer metastasis, very little has been established regarding why it generally takes years for a cancer to become metastatic, knowing that the expression levels of SA synthesis and transferase genes do not go up very substantially (Figure 1). Our model, in conjunction with Tse et al.'s model, suggests that the gradual accumulation of the SA-associated negative charges on cell surface will activate the migration program as discussed in Tse et al. (11) once their electrostatic repulsion reaches a critical point; (2) very little has been established in the literature regarding why certain cancer types metastasize easily and early while other cancer types are less likely to metastasize—our model suggests that it is the combination of the rates of SA production, degradation and transfer to cell surface glycan that determines when a cancer starts to migrate; (3) gangliosides of certain types such as GM2 and GD2 have long been found to be associated with cancer metastasis and the current literature suggests that it is their signaling roles that may be relevant to migration (17); our model suggests that two factors may contribute to the selection of specific types of gangliosides, namely: (i) the number of SAs that can be put on gangliosides per cell, which is determined by the packing density of individual ganglioside types inside the plasma membrane and the number of SAs that each ganglioside of the type can harbor; and (ii) the number of protons produced by the synthesis process of individual ganglioside types: more complex gangliosides produce more protons through their synthesis process per molecule but result in their lower packing densities inside the plasma membrane, hence possibly giving rise to a lower number of total protons per cell. Hence we postulate that the selected ganglioside types are the result of tradeoff between these two factors, which ultimately enables the maximum number of protons to be produced through this combination.

Clearly, our model is a statistical model. We plan to develop a more physics-based model that will allow us to estimate the actual density-level changes as a cancer evolves as well as to calculate the level of electrostatic repulsion in a realistic media environment, hence enabling us to accurately estimate the shield effect of the electrons.

## Methods

### Data

Cancer survival data: The data given in Table 5 are collected from the NCI TCGA website (https://portal.gdc.cancer.gov), which provide clear and detailed clinical information of each cancer patient.

**Table 5:** Cancer types and their transcriptomic data used in this study.

**Cancer type**	**#Tumor samples**	**#Control samples**
Bladder urothelial carcinoma (BLCA)	414	19
Breast invasive carcinoma (BRCA)	1,109	113
Colon adenocarcinoma (COAD)	480	41
Head and Neck squamous cell carcinoma (HNSC)	502	44
Lung adenocarcinoma (LUAD)	535	59
Lung squamous cell carcinoma (LUSC)	502	49
Stomach adenocarcinoma (STAD)	375	32
Pancreatic adenocarcinoma (PAAD)	178	4

Transcriptomics data: We have used all the transcriptomic data of eight cancer types: BLCA, BRCA, COAD, HNSC, LUAD, LUSC, STAD, and PAAD in the TCGA database. Table 6 summarizes the relevant information. Here we used eight cancer types instead of the 14 cancer types we typically use in our recent studies (8, 9, 23) is that we have used TNM staging scheme rather than the more traditional staging approach: stage 1, 2, 3, and 4 since a focus of the study is on the stage of metastasis. For this the TNM staging is clearly more appropriate.

**Table 6:** Regression results for the regulation of key SA genes by transcription factors (TF) and DNA methylation (MT).

**Cancer type**	**Stage**	**NEU1**			**ST3GAL1**			**ST3GAL5**			**CMAS**		
		***R^**2**^***	**MT**	**TF**	***R^**2**^***	**MT**	**TF**	***R*^**2**^**	**MT**	**TF**	***R^**2**^***	**MT**	**TF**
BLCA	Normal	0.98	0.0%	100.0%	0.66	0.0%	100.0%	1.00	79.6%	20.4%	–	–	–
BLCA	Stage II	0.51	12.7%	87.3%	0.77	48.0%	52.0%	0.89	46.3%	53.7%	0.85	10.7%	89.3%
BLCA	Stage III	0.72	44.5%	55.5%	0.70	51.0%	49.0%	0.72	39.2%	60.8%	0.75	32.6%	67.4%
BLCA	Stage IV	0.92	33.9%	66.1%	0.88	52.1%	47.9%	0.65	33.8%	66.2%	0.79	36.5%	63.5%
BRCA	Normal	0.80	0.0%	100.0%	0.96	28.9%	71.1%	0.71	0.0%	100.0%	0.92	1.2%	98.8%
BRCA	Stage I	0.76	48.1%	51.9%	0.83	74.4%	25.6%	0.64	13.5%	86.5%	0.47	0.0%	100.0%
BRCA	Stage II	0.48	21.0%	79.0%	0.63	62.3%	37.7%	0.40	40.1%	59.9%	0.60	37.4%	62.6%
BRCA	Stage III	0.37	44.8%	55.2%	0.70	61.0%	39.0%	0.63	19.9%	80.1%	0.68	13.3%	86.7%
COAD	Normal	0.91	47.6%	52.4%	0.76	0.0%	100.0%	1.00	14.1%	85.9%	0.96	21.8%	78.2%
COAD	Stage I	0.87	58.1%	41.9%	0.72	86.8%	13.2%	0.88	13.9%	86.1%	0.78	49.2%	50.8%
COAD	Stage II	0.69	52.9%	47.1%	0.73	64.4%	35.6%	0.77	30.5%	69.5%	0.82	21.4%	78.6%
COAD	Stage III	0.95	32.9%	67.1%	0.78	48.8%	51.2%	0.69	26.4%	73.6%	0.60	28.7%	71.3%
COAD	Stage IV	0.98	38.0%	62.0%	0.37	100.0%	0.0%	0.85	26.0%	74.0%	–	–	–
HNSC	Normal	1.00	53.1%	46.9%	0.96	89.6%	10.4%	0.96	46.4%	53.6%	0.84	24.9%	75.1%
HNSC	Stage I	1.00	85.9%	14.1%	0.88	68.1%	31.9%	0.96	30.8%	69.2%	0.24	100.0%	0.0%
HNSC	Stage II	0.43	57.9%	42.1%	0.66	49.2%	50.8%	0.91	39.4%	60.6%	0.84	32.4%	67.6%
HNSC	Stage III	0.84	80.3%	19.7%	0.73	37.9%	62.1%	0.84	30.7%	69.3%	0.27	100.0%	0.0%
HNSC	Stage IV	0.73	15.2%	84.8%	0.83	57.9%	42.1%	0.58	26.5%	73.5%	0.83	33.1%	66.9%
LUAD	Normal	1.00	61.5%	38.5%	1.00	43.1%	56.9%	1.00	13.7%	86.3%	0.84	0.0%	100.0%
LUAD	Stage I	0.82	42.2%	57.8%	0.76	61.5%	38.5%	0.68	33.7%	66.3%	0.67	18.5%	81.5%
LUAD	Stage II	0.88	38.4%	61.6%	0.57	41.7%	58.3%	0.67	34.9%	65.1%	0.82	15.7%	84.3%
LUAD	Stage III	0.75	82.2%	17.8%	0.90	67.8%	32.2%	0.84	34.8%	65.2%	1.00	25.3%	74.7%
LUAD	Stage IV	–	–	–	1.00	73.0%	27.0%	0.97	84.1%	15.9%	0.49	0.0%	100.0%
LUSC	Normal	–	–	–	0.95	0.0%	100.0%	1.00	0.0%	100.0%	1.00	0.0%	100.0%
LUSC	Stage I	0.84	37.5%	62.5%	0.75	59.5%	40.5%	0.79	27.6%	72.4%	0.86	13.8%	86.2%
LUSC	Stage II	0.26	37.8%	62.2%	0.73	38.3%	61.7%	0.54	59.5%	40.5%	0.52	30.1%	69.9%
LUSC	Stage III	0.64	65.4%	34.6%	0.62	73.3%	26.7%	0.84	41.3%	58.7%	–	–	–
PAAD	Normal	0.98	0.0%	100.0%	1.00	100.0%	0.0%	1.00	0.0%	100.0%	1.00	0.0%	100.0%
PAAD	Stage I	0.78	83.8%	16.2%	0.98	75.1%	24.9%	–	–	–	0.96	3.2%	96.8%
PAAD	Stage II	0.73	48.1%	51.9%	0.78	36.4%	63.6%	0.61	32.6%	67.4%	0.79	8.3%	91.7%
STAD	Stage I	0.44	0.0%	100.0%	0.57	59.1%	40.9%	0.79	34.6%	65.4%	0.47	78.0%	22.0%
STAD	Stage II	0.92	82.8%	17.2%	0.58	49.5%	50.5%	0.86	38.4%	61.6%	0.86	9.6%	90.4%
STAD	Stage III	0.90	58.3%	41.7%	0.54	81.7%	18.3%	0.77	9.2%	90.8%	0.76	62.9%	37.1%
STAD	Stage IV	1.00	78.0%	22.0%	0.84	69.4%	30.6%	0.98	51.3%	48.7%	0.99	13.2%	86.8%

*Groups with fewer than 20 samples were dropped and marked using “–”. The percentage shown in columns MT and TF are relative sum of squares (see section Methods)*.

Genomics data: Somatic mutation and copy number variation data in TCGA were collected for each of the four SA genes from the UCSC Xena platform (24). Gene-level non-silent mutations calls and copy number variation estimates are used in our study.

DNA methylation data: The beta values of the Illumina Human Methylation 450K platform were collected from the UCSC Xena platform. Probes in the gene body, first exon, UTRs or within 1,500 bp upstream the transcription start site for each of the four SA genes were used.

Transcription factor data: ChIP-Seq validated transcription factor-target gene pairs for each of the four SA genes were collected from the ENCODE database (25), TRRUST database (26), and Marbach et al. (27).

### Prediction of Regulatory Mechanisms via Regression Analysis

For each of the four SA genes, samples with somatic mutations or copy number variations in the gene were filtered out as we're interested in how the relevant transcription factors and DNA methylation co-regulate the expression of the gene.

TPM values for gene expression and M values for DNA methylation were first centered and scaled to have mean 0.0 and standard deviation 1.0. For each target gene g, our goal is to find real values: μ, {α_*i*_} and {β_*j*_} so that the following function is minimized

$$\begin{array}{l} {\left(\text{g}-\left(\mu +\overset{\text{p}}{\underset{\text{i}=1}{\sum }}\alpha_{\text{i}}{\text{x}}_{\text{e}\text{i}}+\overset{\text{q}}{\underset{\text{j}=1}{\sum }}\beta_{\text{j}}{\text{x}}_{\text{m}\text{j}}\right)\right)}^{2}\; \end{array}$$

where *g* is the expression level of the target gene g, *x*_*e*1_, ⋯ , *x*_*ep*_*a*re the expression levels of the transcription factors that regulate g, and *x*_*m*1_, ⋯ , *x*_*mq*_ are the DNA methylation levels of the probes in or around g in the genome. The least squares method was used to solve the optimization problem with Lasso penalty using the R package glmnet (28).

The analysis of variance table was then computed using ANOVA to get the sum of squares (SS) for each parameter. The SS for groups *x*_*e*_ and *x*_*m*_ were then summed up to get *SS*_*TF*_ and *SS*_*MT*_ for contributions by transcription factors and DNA methylations, respectively. $\frac{SS_{MT}}{SS_{TF}+SS_{MT}}$ is used to estimate the level of contribution to transcription regulation by DNA methylation of target gene g.

### Prediction of Concentrations of Gangliosides Based on Gene-Expression Data

We have predicted the relative concentrations of all the gangliosides based on gene expression levels of the relevant enzymes. A key simplifying assumption is: the K_cat_ values for all the enzymes involved in this metabolism are approximately the same since we do not have these values. For each metabolite G, we calculate its total influx and efflux as

$$\begin{array}{l} \sum_{j}G_{j}^{i}\times E\left(g_{j}\right)\;and\;\sum_{k}G_{k}^{o}\times E\left(g_{k}\right) \end{array}$$

where $G_{j}^{i}$ -> G is the jth influx reaction and G -> $G_{k}^{o}$ is the kth efflux reaction of G; and E(*g*_*j*_) is the expression level of gene *g*_*j*_ whose enzyme catalyzes the jth reaction. An iterative procedure is employed to estimate these two quantities till the system converges on {$\left\{G_{j}^{i,o}\right\}$ values. Then we predict G is intracellularly accumulated if $\sum_{j}G_{j}^{i}\times E\left(g_{j}\right)$ is significantly higher than $\sum_{k}G_{k}^{o}\times E\left(g_{k}\right),$ hence used in the plasma membrane. Two levels of “significance” is used: high and moderate in Table 1.

## Data Availability Statement

Publicly available datasets were analyzed in this study. This data can be found here: TCGA, https://portal.gdc.cancer.gov; UCSCXena, https://xena.ucsc.edu; ENCODE, https://www.encodeproject.org; TRRUST, https://www.grnpedia.org/trrust/.

## Author Contributions

YX designed the research. HS, YZ, and HJ performed research. HS, YZ, and YX analyzed data and wrote the paper.

### Conflict of Interest

The authors declare that the research was conducted in the absence of any commercial or financial relationships that could be construed as a potential conflict of interest.
