# Supplementary material for: Elucidation of Functional Roles of Sialic Acids in Cancer Migration
Source: Front Oncol. 2020 Mar 31;10:401. doi: 10.3389/fonc.2020.00401 (PMC7137995; doi:10.3389/fonc.2020.00401)
Supplement: Supplementary file 1 [file Data_Sheet_1.DOCX]

**SUPPLEMENTARY MATERIAL**

**Supplementary Figures**

**Figure S1**: Heat-map showing levels of RMs for selected RMs across 14 cancer types. (Each entry is log2 of fold-change of signature genes. the x-axis is for cancer types; and the y-axis is for reprogrammed metabolic pathways. The level of the Warburg effect is estimated using the fraction of the glycolytic flux going into the TCA cycle, measured using the ratio between the expressions of PDHB and PKM genes; hence the smaller the ratio, the higher the Warburg effect (adapted from ^7^).

**
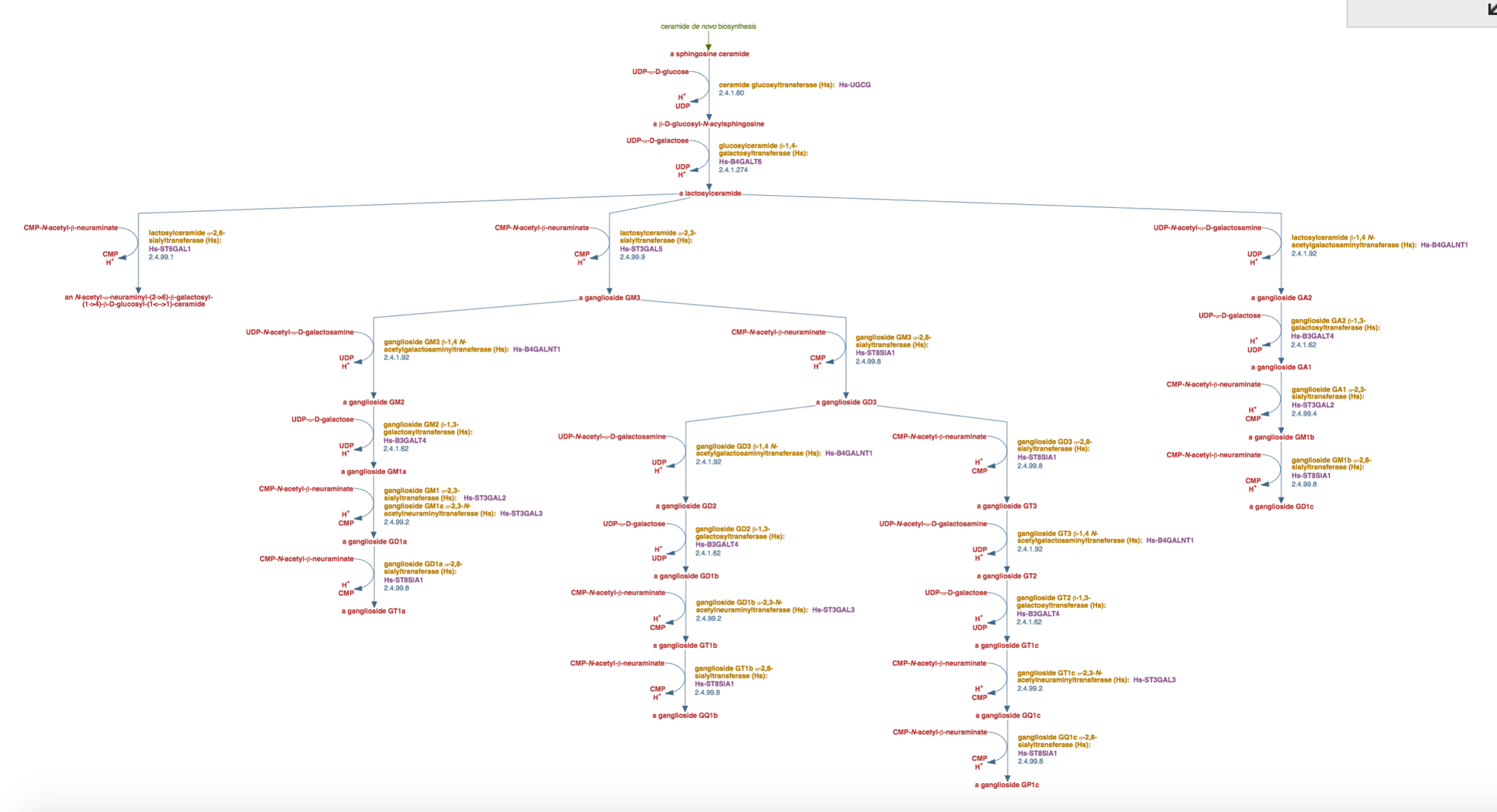
**

**Figure S2:** Metabolic network of ganglioside synthesis (adapted from Biocyc).
